# Observation of strain effect on the suspended graphene by polarized Raman spectroscopy

**DOI:** 10.1186/1556-276X-7-533

**Published:** 2012-09-26

**Authors:** Cheng-Wen Huang, Bing-Jie Lin, Hsing-Ying Lin, Chen-Han Huang, Fu-Yu Shih, Wei-Hua Wang, Chih-Yi Liu, Hsiang-Chen Chui

**Affiliations:** 1 Department of Photonics, National Cheng Kung University, Tainan 70101, Taiwan; 2 Center for Nano Bio-Detection, National Chung Cheng University, Chiayi 621, Taiwan; 3 Institute of Atomic and Molecular Sciences, Academia Sinica, Taipei 10617, Taiwan; 4 Advanced Optoelectronic Technology Center, National Cheng Kung University, Tainan 70101, Taiwan

**Keywords:** Graphene strain, Polarization Raman spectroscopy, suspended graphene, 78.67.Wj (optical properties of graphene), 74.25.nd (Raman and optical spectroscopy), 63.22.Rc (phonons in graphene)

## Abstract

We report the strain effect of suspended graphene prepared by micromechanical method. Under a fixed measurement orientation of scattered light, the position of the 2D peaks changes with incident polarization directions. This phenomenon is explained by a proposed mode in which the peak is effectively contributed by an unstrained and two uniaxial-strained sub-areas. The two axes are tensile strain. Compared to the unstrained sub-mode frequency of 2,672 cm^−1^, the tension causes a red shift. The 2D peak variation originates in that the three effective sub-modes correlate with the light polarization through different relations. We develop a method to quantitatively analyze the positions, intensities, and polarization dependences of the three sub-peaks. The analysis reflects the local strain, which changes with detected area of the graphene film. The measurement can be extended to detect the strain distribution of the film and, thus, is a promising technology on graphene characterization.

## Background

Raman and surface-enhanced Raman spectroscopy have been widely used to investigate vibration properties of materials [1-6]. Recently, they have been used as powerful technologies to characterize the phonons of graphene [7-14]. With its one to several atomic layers, graphene is the thinnest *sp*^2^ allotrope of carbon, and therefore holds many unique electrical and optical properties, interesting scientists and technologists [15-19]. The unique properties change with the number of atomic layers, defects, and dopants. These factors also affect graphene's phonon modes, and therefore, Raman spectroscopy is a useful method to reflect the variation of the properties [20-22]. In addition, Raman spectroscopy can be employed to determine strain, which modifies the characteristic of materials, such as band structure, and thus influences the performance of corresponding devices [23-25]. Recently, several groups study strain of graphene by artificially bending or stretching the film and then measuring the corresponding Raman spectra [26-30].

## Methods

**Figure 1:**
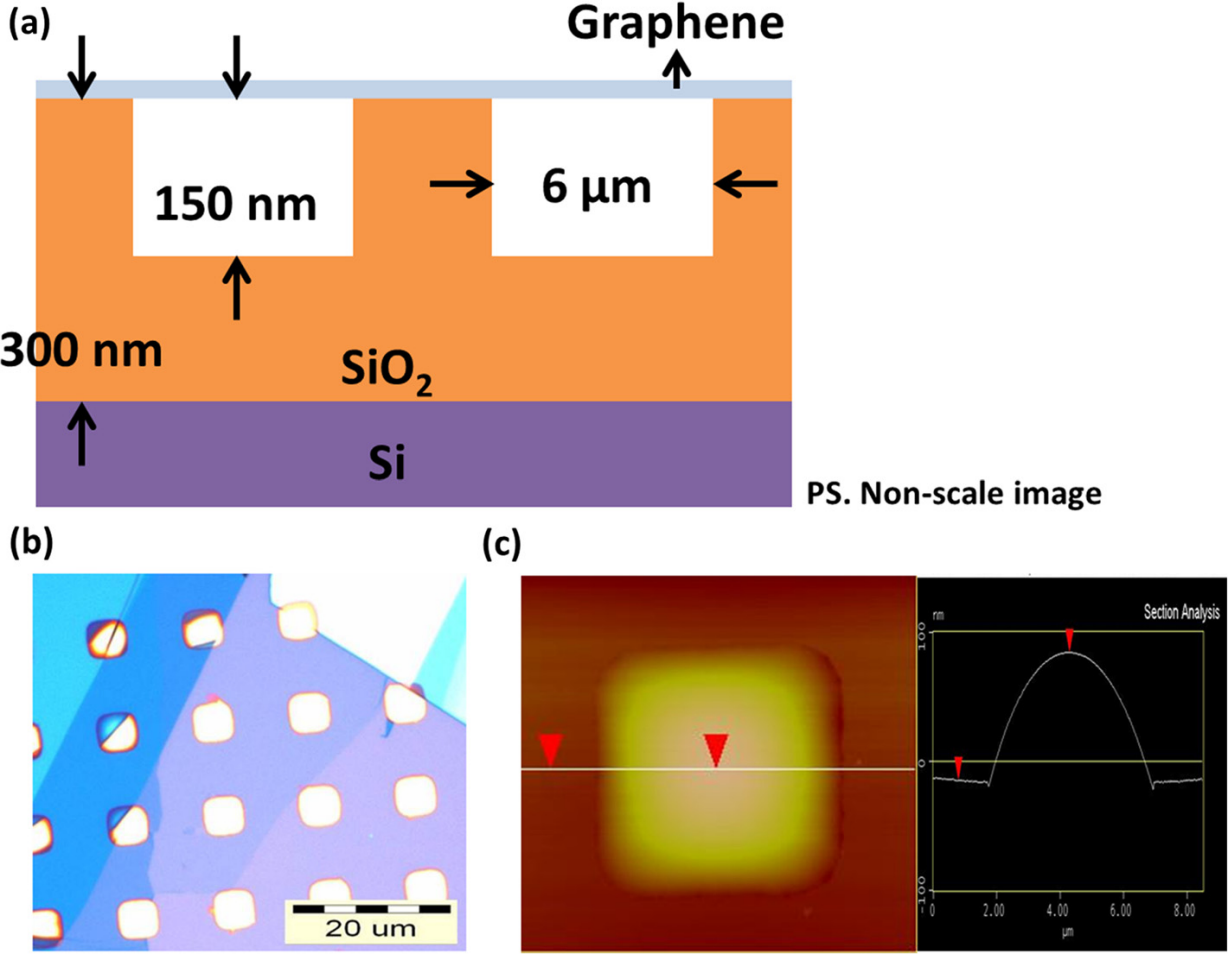
**Images of the suspended graphene.** (**a**) The scheme, (**b**) optical image, and (**c**) AFM image of the suspended graphene. The AFM image included the topography and its cross-section.

Suspended graphene are fabricated by mechanical exfoliation of graphene flakes onto the oxidized silicon wafer. First, ordered squares with areas of 6 μm^2^ are defined by photolithography on an oxidized silicon wafer with oxide thickness of 300 nm. Reactive ion etching is then used to etch the squares to a depth of 150 nm. Micromechanical cleavage of HOPG with scotch tape is then used to deposit the suspended graphene flakes over the indents, as shown in the schematic of Figure 1a. Optical micrograph and atomic force microscopy (AFM) image, as shown in Figure 1b,c, were used to characterize the suspended graphene. The surface of suspended graphene was bulging as indicated by AFM cross-section. The strain and defects of graphene are usually measured by Raman spectroscopy. To understand the strain of the suspended graphene, a micro-Raman microscope was used to perform Raman polarization-dependence measurements. A 532-nm frequency-doubling Nd-YAG laser serves as the excitation light source. The polarization and power of the incident light were adjusted by a half-wave plate and a polarizer. The laser power was monitored by a power meter and maintained through these measurements. The excitation laser power measured by the power meter is 8 mW. The laser power is measured on the delivered path between the polarizer and spectroscopy. After the delivery of laser light, the power of laser on the graphene surface is finally about 0.45 mW. The laser beam was focused by a ×50 objective lens (NA = 0.75) to the sample with a focal spot size of approximately 0.5 μm, representing the spatial resolution of the Raman system. The scattered radiation was collected backward with the same objective lens and polarization-selected by a polarization analyzer. Finally, the radiation was sent to a 55-cm spectrometer plus a liquid-nitrogen-cooled charge-coupled device for spectral recording. For the polarization dependent measurement, the polarization of incident (scattered) laser is controlled by the polarizer (analyzer). In the exploration, the polarization direction of the incident and scattered light are variable and fixed, respectively. The variability of polarization direction of the scattered light is 20°.

## Results and discussion

**Figure 2:**
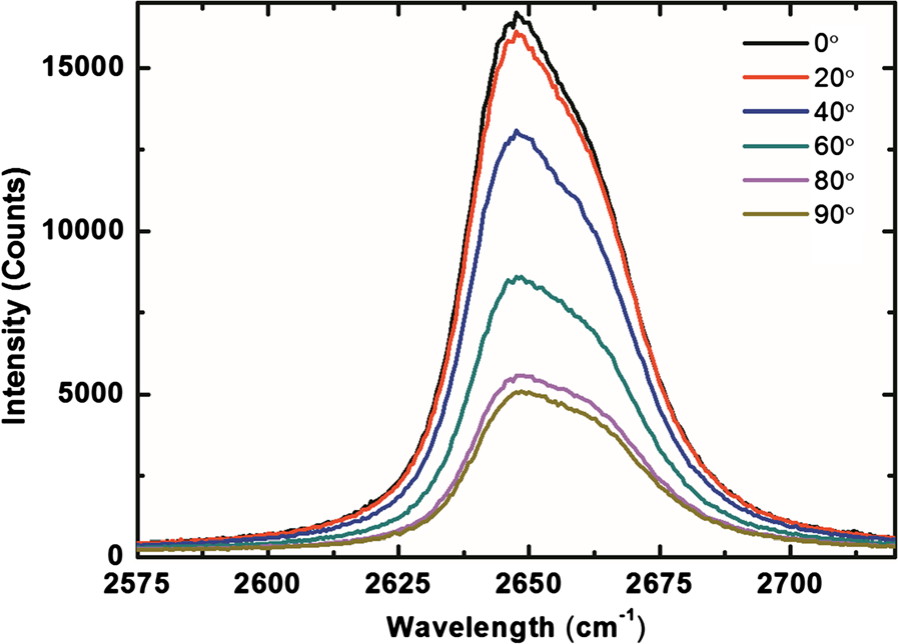
**Polarized Raman spectra of 2D modes under incident lights with different polarization angle (*Φ*).** The *Φ* is defined as the included angle between the incident polarization direction and the analyzer.

Polarized Raman spectra of 2D modes under incident lights with different polarization angle (*Φ*) are shown in Figure 2. The *Φ* is defined as the included angle between the incident polarization direction and the analyzer. The clear anti-symmetric spectra with different polarization angle can be observed in the spectra. Fitting of the 2D peaks is done using a double-Lorentzian function for *Φ* of (a) 0° and (b) 90°.

**Figure 3:**
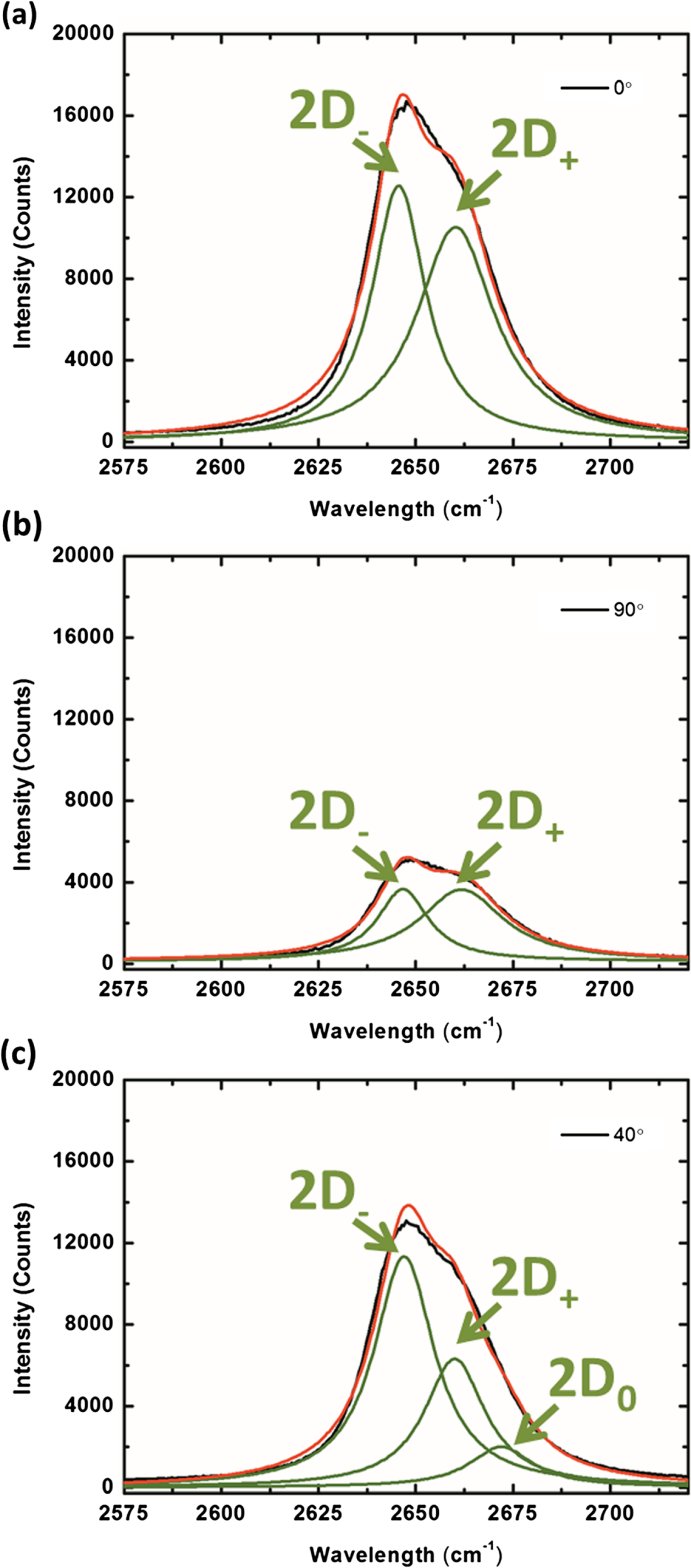
**Fitting the 2D peaks with a double-Lorentzian function.***Φ* of (**a**) 0° and (**b**) 90°. The two angles relate to the two maximum peak positions in Figure 2, respectively. The two Lorentz peaks for (a) are at 2,646 (2D_−_) and 2,660 (2D_+_) cm^−1^, while that for (b) are at 2,647 (2D_−_) and 2,662 (2D_+_) cm^−1^. (**c**) For *Φ* of 40°, fitting the 2D peak of graphene by a triple-Lorentzian function whose three peaks correspond to the sub-modes of 2D_+_, 2D_−_, and 2D_0_. The green curves represent the fitting peaks for the corresponding spectra. The black curves display the spectra, while the red ones show the profiles by adding all the related fitting peaks.

The 2D peak originates from four-step Stokes-Stokes double-resonance Raman scattering [31]. According to the previous review, the 2D band split when strain is applied [32]. The two angles relating to the two maximum peak positions are exhibited in Figure 3, respectively. The two Lorentzian peaks for Figure 3a are at 2,646 (2D_−_) and 2,660 (2D_+_) cm^−1^, while that for Figure 3b are at 2,647 (2D_−_) and 2,662 (2D_+_) cm^−1^. To systematically analyze the sub-peak of the 2D modes, the spectra of 2D modes with different polarization angle can be fitted by double-Lorentz function, and the 2,647 and 2,660 cm^−1^ by average of all the peak positions of 2D_+_ and 2D_−_ modes with different polarization angles. The 2D_0_ showed the peak position at 2,672 cm^−1^ as an original 2D peak in the unstrained graphene. The peak positions of 2D_+_ and 2D_−_ modes compared with an original 2D peak both red shifts. The results showed that the 2D_+_ and 2D_−_ modes are both tensile strains on suspended graphene. To understand the strain effect of the suspended graphene, fitting the 2D peak of graphene by a triple-Lorentzian function whose three peaks correspond to the sub-modes of 2D_+_, 2D_−_, and 2D_0_ with *Φ* = 40°, which is just an example of all the spectra, is shown in Figure 3c.

**Figure 4:**
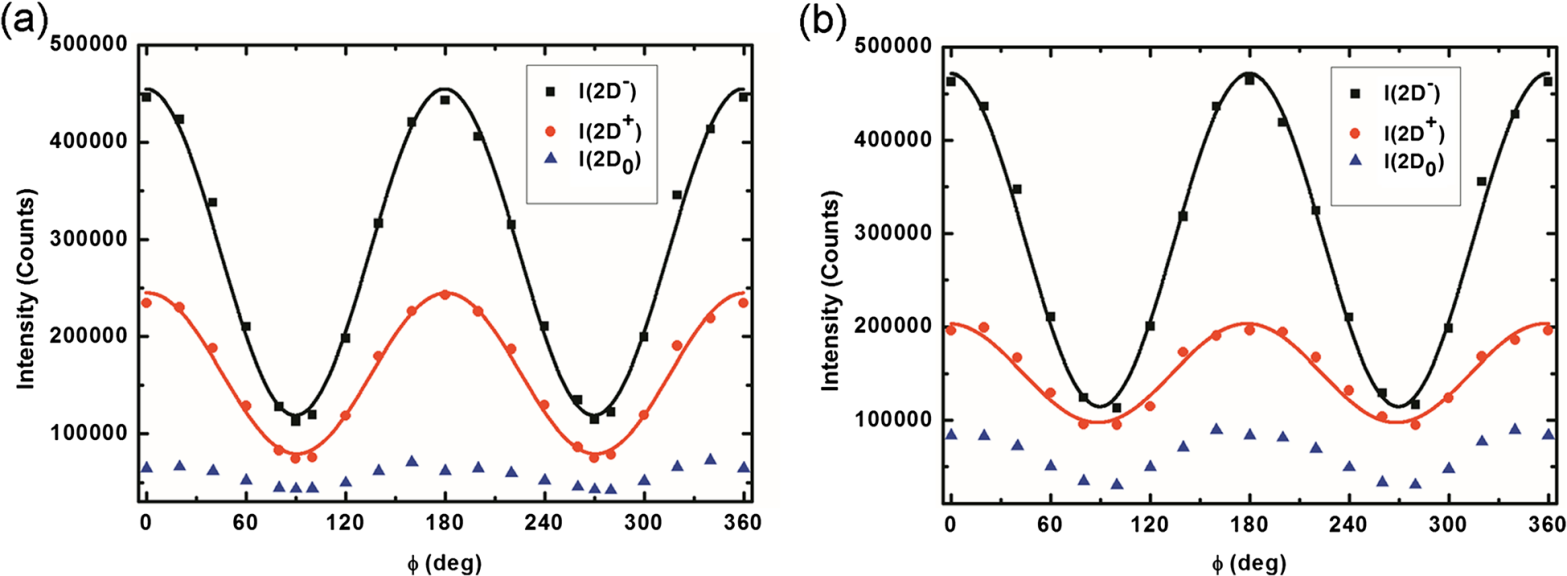
**Analysis of intensity.** (**a**) Suspended and (**b**) supported graphenes. The plots of *I*(2D_+_), *I*(2D_−_), and *I*(2D_0_) as functions of *Φ*. The symbol ‘*I*’ denotes the intensity of the corresponding sub-peak obtained by fitting the related Raman spectrum with a triple-Lorentzian function. The obtained intensities are shown by the dots, which are fitted by the form of *A*cos^2^(*Φ* − *Φ*_0_) for *I*(2D_+_) and *I*(2D_−_), and of a constant of *A*, where *A* and *Φ*_0_ are fitting parameters. The black and red lines display the fitting results.

To systematically analyze the intensities of the fitting sub-peaks, the plots of *I*(2D_+_), *I*(2D_−_), and *I*(2D_0_) as functions of *Φ* is shown in Figure 4. The analysis of the suspended graphene was shown in Figure 4a. The 2D_+_ and 2D_−_ sub-bands which have the peak positions of 2,647 and 2,660 cm^−1^, respectively, showed a prominent sinusoidal intensity modulation with a period of 180°. Both the modulation of the 2D_+_ and 2D_−_ bands can be fitted by a function of cos^2^(*θ*_A_ − *θ*_P_), where *θ*_A_ and *θ*_P_ are the polarization angles of the analyzer and polarizer, respectively. Both the intensities of 2D_+_ and 2D_−_ modes are the maximum when *Φ* = 0° and minimum when *Φ* = 90°. This result of suspended graphene is very different from those of the supported graphene by previous research [32]. Compared with supported graphene, the same direction of tensile strain on the suspended graphene by analyzing 2D_+_ and 2D_−_ modes can be obtained.

The strains of the 2D_+_ and 2D_−_ can be calculated by employing the Grüneisen parameter [27] (γ) for the 2D mode of graphene. The corresponding equation is written as $\omega =\omega_{0}\gamma \left(\varepsilon_{\text{xx}}+\varepsilon_{\text{yy}}\right)$, where ω_0_ is the 2D peak position at zero strain and Δω is the shift caused by the strain of ε. For the uniaxial strain, $\varepsilon_{\text{xx}}$is the uniaxial strain and $\varepsilon_{\text{yy}}=-0.186\varepsilon_{\text{xx}}$ is the relative strain in the perpendicular direction due to Poissons’ ratio of graphene [33]. In addition, the γ has been measured as 1.24 from the experiment on CNTs [34]. Hence the stains of $\varepsilon_{\text{xx}}$ for the 2D_+_ and 2D_−_ are estimated as 0.44% and 0.93%, respectively. Based on these results, the distribution of strain on the suspended graphene can be obtained through our analysis.

Another interesting phenomenon measured from our sample can be observed in Figure 4b. The analysis of the supported graphene which was used the same method in Figure 4a was shown in Figure 4b. The 2D_+_ and 2D_−_ sub-bands having the peak positions of 2,651 and 2,661 cm^−1^, respectively, showed a prominent sinusoidal intensity modulation with a period of 180°. Both the modulation of the 2D_+_ and 2D_−_ bands can be fitted by a function of cos^2^(*θ*_A_ − *θ*_P_), where *θ*_A_ and *θ*_P_ are the polarization angles of the analyzer and polarizer, respectively. Both the intensities of 2D_+_ and 2D_−_ modes are the maximum when *Φ* = 1° and minimum when *Φ* = 91°. Using the same calculation, the stains of $\varepsilon_{\text{xx}}$ for the 2D_+_ and 2D_−_ are estimated as 0.44% and 0.93%, respectively. The result in Figure 4b is similar with Figure 4a. Based on the results, we believed the strain will be relaxed to a new condition during the fabricated process of substrate.

## Conclusion

We have explored the suspended graphene by polarized Raman spectroscopy. In the exploration, the polarization direction of the incident and scattered light are variable and fixed, respectively. The position and intensity of the graphene's 2D peak is modified by the incident polarization, and the modification is explained by a proposed biaxial-strained model. In this model, the 2D peak is contributed by three effective areas related to unstrained and two tensile-strained graphene, respectively. The two strains are uniaxial and in the same directions. The strength of the strains is quantified through our analysis. This analytical method can be used to probe strain and help us understand the situation of suspended graphene. Hence, this method provides great application potential on graphene-based electrical and optical devices, whose performance usually relies on strain.

## Competing interests

The authors declare that they have no competing interests.

## Authors' contributions

CWH and BJL carried the experimental parts: the acquisition, analysis, and interpretation of data. CWH also had been involved in drafting the manuscript. HYL and CHH performed the analysis and interpretation of data. They also had been involved in revising the manuscript. FYS and WHW (Institute of Atomic and Molecular Sciences, Academia Sinica) prepared the samples, suspended the graphene using micromechanical method, and captured the OM and AFM images. CYL has made substantial contributions to the conception and design of the study, and the critical revision of the manuscript for important intellectual content. HCC, the corresponding author, had made substantial contributions to the conception and design of the study, and had been involved in drafting the manuscript and revising it critically for important intellectual content. All authors read and approved the final manuscript.

## Authors' information

CWH received his BS degree in Electronic Engineering from the National University of Kaohsiung, Kaohsiung, Taiwan, in 2008. He studied his MS degree in 2008 and PhD degree immediately in 2009. Currently, he is a PhD candidate in the Department of Photonics, National Cheng Kung University, Tainan, Taiwan. He focuses on the property of graphene and surface plasmon resonance of nanoparticles.

BJL received his BS degree in Physics from the National Chung-Hsing University, Taichung, Taiwan, in 2010. He received his MS degree in Photonics from the National Cheng Kung University, Tainan, Taiwan, in 2012. His research interests mainly include Raman measurement of graphene. He is in compulsory military service now.
